# A method for MRI‐guided bronchoscopy to identify obstructed airway segments

**DOI:** 10.14814/phy2.70119

**Published:** 2025-02-20

**Authors:** David G. Mummy, Marrissa J. McIntosh, Katherine J. Carey, Shannon Kehoe, Stephane Esnault, Mats W. Johansson, Michael D. Evans, Ronald L. Sorkness, Mark Schiebler, Nizar N. Jarjour, Loren C. Denlinger, Sean B. Fain

**Affiliations:** ^1^ Department of Radiology Duke University Medical Center Durham North Carolina USA; ^2^ Department of Radiology University of Iowa Iowa City Iowa USA; ^3^ Department of Medical Physics University of Wisconsin—Madison Madison Wisconsin USA; ^4^ Department of Radiology University of Wisconsin—Madison Madison Wisconsin USA; ^5^ Division of Allergy, Pulmonary and Critical Care Medicine, Department of Medicine University of Wisconsin—Madison Madison Wisconsin USA; ^6^ Institute for Translational Research in Inflammation—U1286—INFINITE University of Lille Lille France; ^7^ Clinical and Translational Science Institute University of Minnesota Minneapolis Minnesota USA; ^8^ Roy. J Carver Department of Biomedical Engineering University of Iowa Iowa City Iowa USA

**Keywords:** asthma, bronchial wall biopsy, bronchoalveolar lavage, CT air trapping, hyperpolarized gas MRI

## Abstract

Bronchoscopy is not conventionally guided by prior knowledge of segmental airway obstruction. Hyperpolarized gas magnetic resonance imaging (MRI) ventilation abnormalities and computed tomography (CT) air trapping are related to lung function and asthma severity but have not been used to target segmental inflammation and remodeling. We evaluate the feasibility of using bronchoscopy guided by ^3^He MRI and CT to reveal differences in inflammatory response, morphology, and cellular activity in poorly‐ (defect) versus well‐ventilated (control) lung regions. Eleven participants (5 female; age, 22.8 ± 3.4 years; 9 asthma) who experienced a cold with increased lower airway symptoms underwent ^3^He MRI and/or CT at least 6 weeks after recovery. Differences between defect and control regions were compared. In defect as compared to control sites, bronchoalveolar lavage neutrophils (*p* = 0.06) and granulocytes (*p* = 0.08) trended towards an increase; inflammatory mediators (i.e., 15‐epi‐LXA4, LXA4) were also significantly different (*p* < 0.05) between sites. Correlations were observed between macrophages, neutrophils, and eosinophils with inflammatory mediators (i.e., 15‐epi‐LXA4, LXA4, LTB4). Correlations were observed for macrophages and neutrophils with 15‐epi‐LXA4, and eosinophils with LXA4 and leukotriene B4. Basement membrane wall thickness was similar for defect versus control sites (*p* = 0.9). These results support the feasibility of image‐guided methods to identify airway obstruction phenotypes.

## Graphical abstract

Image guided bronchoscopy using MRI and CT (Methods, left) can identify regions of airway injury (Results, right) to evaluate cellular and molecular phenotypes of asthma.

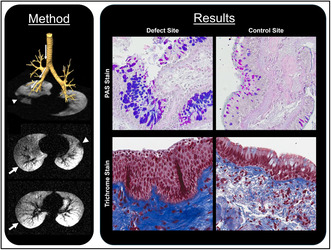

## 1 INTRODUCTION

Investigative bronchoscopic sampling of fluid and tissue has long been used for the in vivo evaluation of cellular activity and airway morphology (Godard et al., 1982; Lozewicz et al., 1990), and has been shown to be well‐tolerated in asthma patients (Moore et al., 2011). Bronchoalveolar lavage (BAL), histochemical (HC) and immunohistochemical (IHC) stains and morphological measures of biopsies obtained via bronchoscopy enable a range of specific measurements which are associated with pathological response (Kelly et al., 2017). However, this technique traditionally takes place without prior knowledge of the presence or absence of airway obstruction within the region undergoing sampling. Evaluating localized cellular and morphological phenomena associated with segmental airway obstruction may provide insights into the pathophysiology of chronic obstructive lung disease and aid in the development of targeted therapies.

Hyperpolarized (HP) gas magnetic resonance imaging (MRI) provides a means of directly visualizing gas distribution in the lungs (Kauczor et al., 1997; Moller et al., 2002) without the use of ionizing radiation. Defect extent is commonly measured as the ventilation defect percentage (VDP) (1), which, in asthma, is spatially heterogeneous (Aysola et al., 2010; Zha et al., 2018), related to computed tomography (CT) structural abnormalities (i.e., air trapping, airway wall thickness) (Carey et al., 2023; Fain et al., 2008; Svenningsen et al., 2018), and associated with and predictive of exacerbations (Mummy et al., 2020; Mummy, Kruger, et al., 2018); ventilation defects have previously been shown to be spatially and quantitatively reproducible over the short (same‐day, 7‐day) (Mathew et al., 2008; Niles et al., 2013) and long‐term (7 years) (Eddy et al., 2019). Moreover, evidence of granulocytic inflammation is highly correlated with both global (Svenningsen et al., 2018) and lobar VDP (Fain et al., 2008).

The purpose of this work was to prospectively use HP ^3^He MRI to obtain paired bronchoscopy samples from ventilation defect and well‐ventilated lung regions within the same subject in a pilot population of patients who had experienced a recent viral‐induced exacerbation. Some of these findings have been previously presented in the form of two abstracts (Mummy et al., 2015; Mummy, Kehoe, et al., 2018).

## 2 MATERIALS AND METHODS

### 2.1 Participants and study design

Participants aged 16–60 years provided written informed consent to the Viral‐Induced Asthma Exacerbation (VIAX) (Denlinger et al., 2011) longitudinal study. This prospective study was compliant with the Health Insurance Portability and Accountability Act (HIPAA) and was approved by the University of Wisconsin Health Sciences Institutional Review Board (IRB H‐2005‐0070). Image‐guided bronchoscopy was performed based on the presence of a defect after recovery from a virus‐induced exacerbation with the intent of sampling regions at the leading edge of airway injury. Participants underwent ^1^H (standard proton) and ^3^He MRI, CT, and spirometry at least 6 weeks post‐viral exacerbation and were excluded if they reported other respiratory complications (i.e., current smoker or significant smoking history (exceeding 5 pack years), pulmonary edema, atelectasis, upper airway obstruction). Bronchoscopy was performed to obtain BAL and bronchial wall biopsy samples within 30 days following imaging.

### 2.2 Image acquisition and analysis

Anatomical ^1^H and HP ^3^He MRI were acquired under breath‐hold conditions by using a 1.5 Tesla Signa HDx scanner (GE Healthcare, Milwaukee WI). ^3^He was polarized using a Polarean IGI 9600 hyperpolarizer (Polarean Inc., Durham NC) and mixed with N_2_ such that the total volume of ^3^He and N_2_ was 14% of the participant's total lung capacity. The gas mixture was prepared and delivered in a Tedlar bag (Jensen Inert Products, Coral Springs FL). Participants were positioned supine with the gas dose inhaled from functional residual capacity.

**FIGURE 1:**
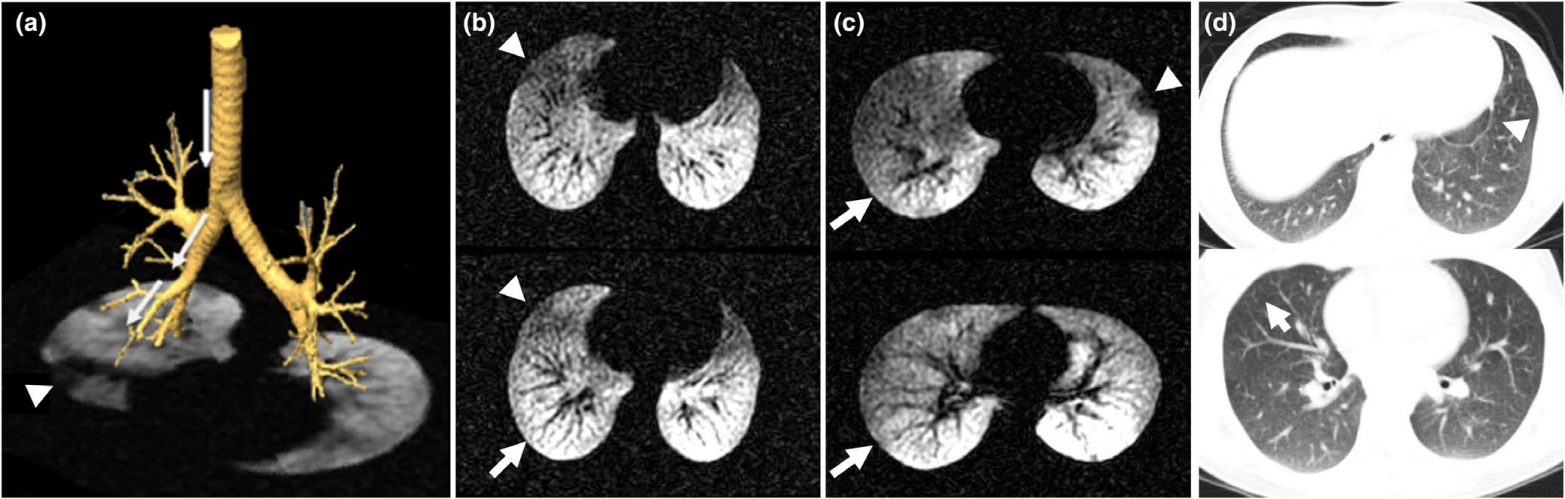
(a) 3D airway tree generated from CT (VIDA Diagnostics, Coralville, IA) superposed on axial hyperpolarized (HP) gas MR. An example path through the airway tree towards an area of ventilation defect (“target”) is shown. (b) MR image‐guided target defect located in basal region of the right middle lobe (arrowheads) and control region in the right lower lobe (arrows) for an MRI case. The case exemplified here is illustrative of the reduced, but not fully obstructed, ventilation regions identified in many participants. (c) MR image‐guided target defect located in anterior left upper lobe (arrowhead) and control region in the right lower lobe (arrows) for an MRI case. This example is illustrative of the typical fully obstructed defect case. (d) CT image‐guided target air trapping defect located in the left lower lobe near the heart (arrowhead) and control region in the right middle lobe (arrow).

Ventilation defects were located anatomically using the corresponding slice from a ^1^H MRI and registered CT reference (Figure 1a). Defects were visually identified as regions of no or low signal on an axial HP ^3^He MRI. Lower but not complete absence of gas signal was still defined as a visual defect in some cases (Figure 1b,c). In a subset of participants where MRI was not available, defects were located by proxy on CT by identifying regions of hyperlucency, indicative of air trapping (Figure 1d). MR images were segmented and quantified using custom software tools implemented in MATLAB (MathWorks, Natick MA) as described previously (Zha et al., 2018). Whole lung VDP was calculated as the ventilation defect volume normalized to the total ^1^H thoracic cavity volume.

Chest CT was performed at end expiration (functional residual capacity) on a 64 slice multi‐detector scanner (VCT 64, GE Healthcare). Effective CT dose was adjusted to mitigate radiation exposure and maintain image quality (Sieren et al., 2016). Air trapping was quantified from CT images as the relative area less than −850 (RA_−850_) Hounsfield units (HU).

### 2.3 Bronchoscopy and analysis

BAL and endobronchial biopsy samples of the airway wall were obtained via image‐guided bronchoscopy at both defect and control sites within the same or contralateral lung of a given participant. BAL samples were analyzed for differential cell counts. Oxylipin mediators contribute to the transition between pro‐inflammatory and pro‐resolution states (Tavares et al., 2023; Townsend et al., 2023) thus, oxylipin levels with and without normalization for total protein concentration in the BAL fluid were also measured. Markers of interest are shown in Table 1 and included lipoxin A4 (LXA4) and 15‐epimer (15‐epi) of LXA4 (inhibit neutrophilic infiltration and production of pro‐inflammatory cytokines and chemokines (Bonnans et al., 2007; Takano et al., 1997)) and leukotriene B4 (induces recruitment and activation of granulocytes and monocytes (Crooks & Stockley, 1998)). Biopsy samples were embedded in paraffin and frozen before being sectioned and mounted on slides (Kelly et al., 2017). Biopsy samples were stained with H&E and reviewed by an expert pulmonary pathologist. A subset of participants was selected for further HC and IHC staining (markers described in Table 2) based on a visual evaluation by the pathologist of the quality of tissue biopsy presentation on H&E stains. Specifically, these samples were chosen based on the quality of the sample and a preference for sections cross‐sectional to the airway.

**TABLE 1:** Oxylipin mediators obtained from bronchoalveolar lavage fluid.

Oxylipin mediator	Supplier/catalogue number
LXA4	Neogen Corporation #407010
LTB4	Cayman Chemical #520111
15‐epi‐LXA4	Neogen Corporation #407110
CysLTs	Cayman Chemical #10009291 and #500390

Abbreviations: 15‐epi, 15‐epimer; CysLTs, cysteinyl leukotrienes; LTB4, leukotriene B4; LXA4, eicosanoid lipoxin A4.

**TABLE 2:** Stains performed on paired biopsy samples in a subset of participants.

Stain	Primarily indicates	Supplier/catalogue number
CD4	Helper T cells	Ventana #790–4423
CD8	Cytotoxic T cells	Ventana #790–4460
CD10	Mature granulocytes	Ventana #790–4506
CD20	B‐cells	Ventana #760–2531
CD68	Monocytes and macrophages	Ventana #790–2931
CD117	Mast cells	Abcam #Ab15580
CD138	Cell proliferation	Cell‐Marque #760–4248
Ki‐67	Cell proliferation	Abcam #Ab15580
Synaptophysin	Neuroendocrine cells	Ventana #790–4460
Trichrome (Masson's)	Connective tissue, nuclei and cytoplasm	Epredia Kit #87019
Alcian Blue/PAS	Mucus	Newcomer Supply #1003

*Note*: Secondary antibody Omni‐Map anti‐Rabbit HRP supplied by Ventana #760–4311 was used for all analyses, except for CD20, CD68 and CD138 which used Ultra‐Map anti‐Mouse HRP supplied by Ventana #760–4313.

Abbreviation: PAS, Periodic Acid‐Schiff.

**FIGURE 2:**
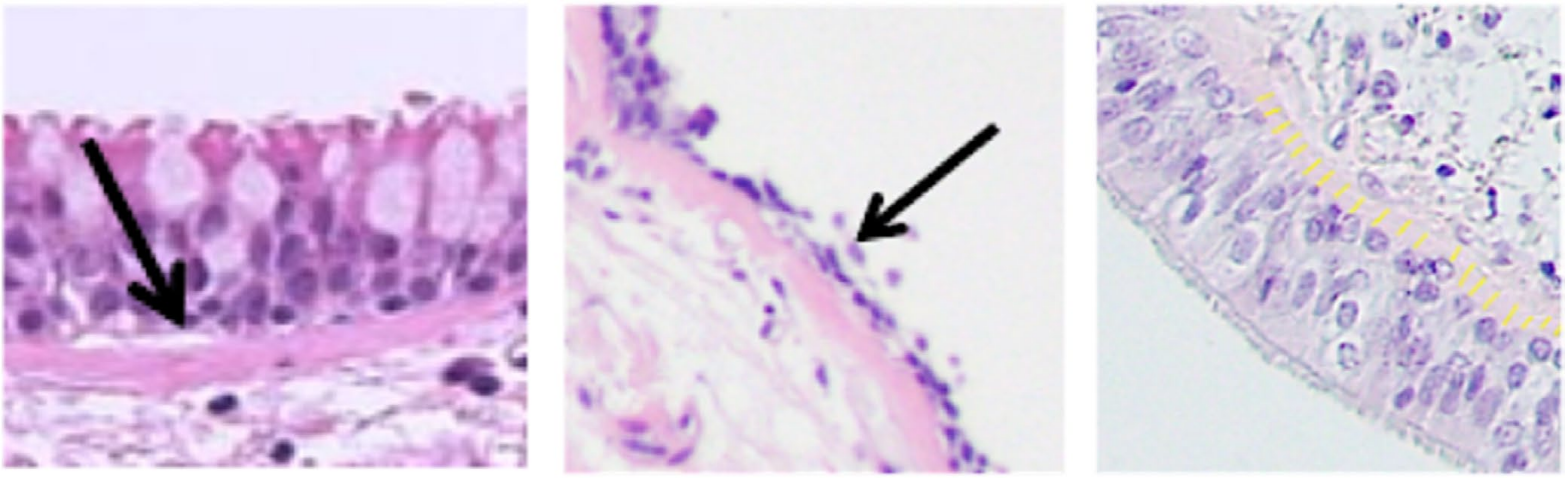
Basement membrane analysis. Basement membrane shown on H&E samples with epithelium (left) and without epithelium (center). Approximately 20 transverse membrane measurements (yellow) were averaged for each data point (right).

Basement membrane thickness was measured on H&E stains using ImageJ software (https://imagej.nih.gov/ij/), as shown in Figure 2, by two imaging scientists (KJC and SK) under pathologist guidance. In each sample, approximately 20 measurements were obtained from areas both with and without epithelium, as available. The measurements were averaged to determine the mean wall thickness for that sample. Image‐based quantitative analysis of HC and IHC stained sections was performed using Aperio ImageScope software (https://www.leicabiosystems.com/digital‐pathology/manage/aperio‐imagescope/). Regions of interest (ROI) of a standard size were defined on H&E stains for each sample. These ROIs were transferred to equivalent areas in HC/IHC stained slides from adjacent sections by the scientist performing image‐based measurements (KJC and SK). Cell counts were then obtained and normalized to total ROI area.

### 2.4 Statistical analysis

Normality was evaluated using Shapiro–Wilk tests, which showed that data was not normally distributed. Differences in BAL fluid cell counts, oxylipin levels as well as basement membrane thickness in defect versus control sites were evaluated using Wilcoxon Signed Rank tests. Univariate relationships were evaluated using Spearman correlations. Observable differences in cell counts for each participant were defined as a factor of two increase or decrease between defect and control sites. Data are presented as the mean ± standard deviation unless otherwise specified. All statistical analysis was performed using SPSS (Version 29.0, IBM). *p* values less than 0.05 were considered statistically significant.

## 3 RESULTS

**FIGURE 3:**
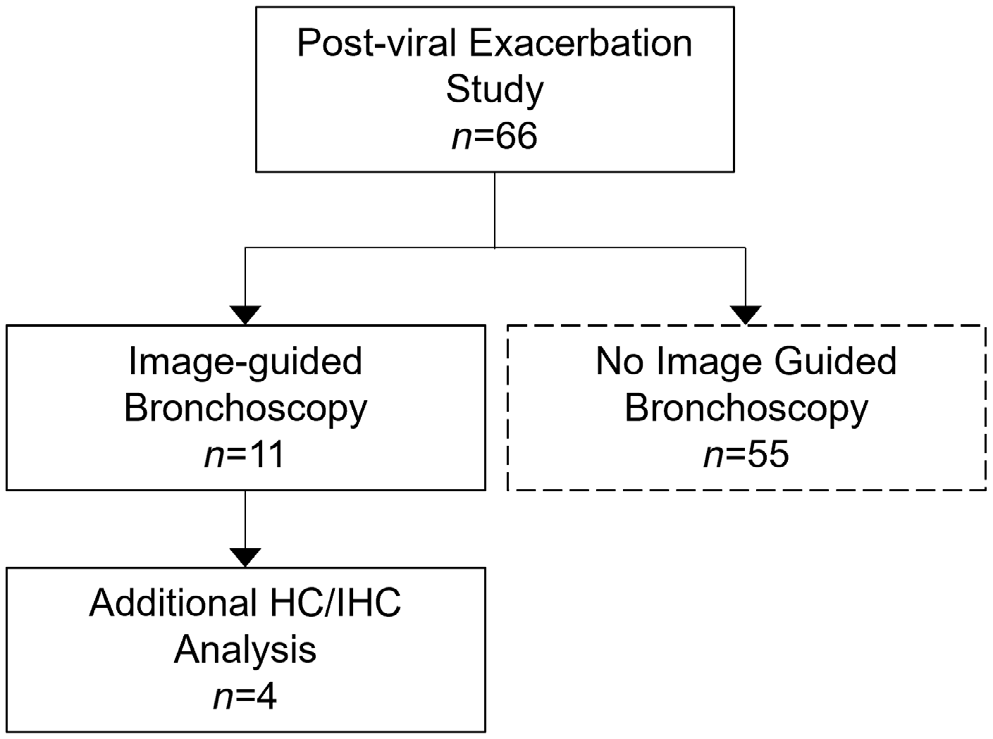
CONSORT Diagram. Of the 66 participants enrolled in the Viral‐Induced Asthma Exacerbation (VIAX) study, a total of 11 participants underwent image‐guided bronchoscopy for BAL and tissue biopsy. Biopsy samples from four participants also underwent extensive histochemical (HC) and immunohistochemical (IHC) analysis. Participants in dashed boxes were not included in this manuscript analysis.

The Consolidated Standards of Reporting Trials diagram in Figure 3 shows that of the 66 participants enrolled in the cold virus‐induced exacerbation study as detailed previously (Denlinger et al., 2011), 11 (5 females, 6 males; 9 asthma; age, 22.8 ± 3.4 years) underwent image‐guided bronchoscopy. Participants in this study were recruited based on having a recent exacerbation due to a detected rhinovirus (*N* = 7) or respiratory syncytial virus (*N* = 2) in the upper airways 6–8 week prior to HP gas MRI. Table 3 provides a summary of participant demographics, pulmonary function, and imaging measurements acquired just prior to image‐guided bronchoscopy. Briefly, the mean forced expiratory volume in the first second (FEV_1_) and FEV_1_ to forced vital capacity ratio (FEV_1_/FVC) were in the normal range, while whole lung VDP (Pike et al., 2015) and RA_−850_ (Mets et al., 2012) measures were typical of those seen in mild asthma or healthy normal participants. Bronchoscopy was performed 11 ± 8 days after imaging (median [min–max] = 9 [2–29] days), which was also 73 ± 21 days since the onset of cold symptoms (median [min–max] = 71 [51–129] days). All 11 participants had BAL performed in both the defect and control segments and 8 had endobronchial biopsies collected. Biopsy samples from four of these participants met quality standards and also underwent HC and IHC analysis.

**TABLE 3:** Demographics, pulmonary function, and imaging measurements.

Parameter	Cohort (*n* = 11)
Age (years)	22.8 ± 3.4
Female Sex *n* (%)	5 (46)
Asthma *n* (%)	9 (82)
FEV_1_ (%_pred_)	101 ± 16
FEV_1_/FVC (%_pred_)	97 ± 14
VDP (%)	1.1 ± 1.5
Defect site	1.1 ± 1.9
Control site	0.9 ± 1.6
RA_−850_ (%)	1.6 ± 1.5
Defect site	4.0 ± 5.8
Control site	1.8 ± 1.8

*Note*: Data is reported as mean ± standard deviation unless indicated otherwise.

Abbreviations: %pred, percent of predicted value; FEV_1_, forced expiratory volume in first second; FVC, forced vital capacity; RA_−850_, relative area of the lung less than −850 Hounsfield Units; VDP, ventilation defect percentage.

### 3.1 BAL results

**FIGURE 4:**
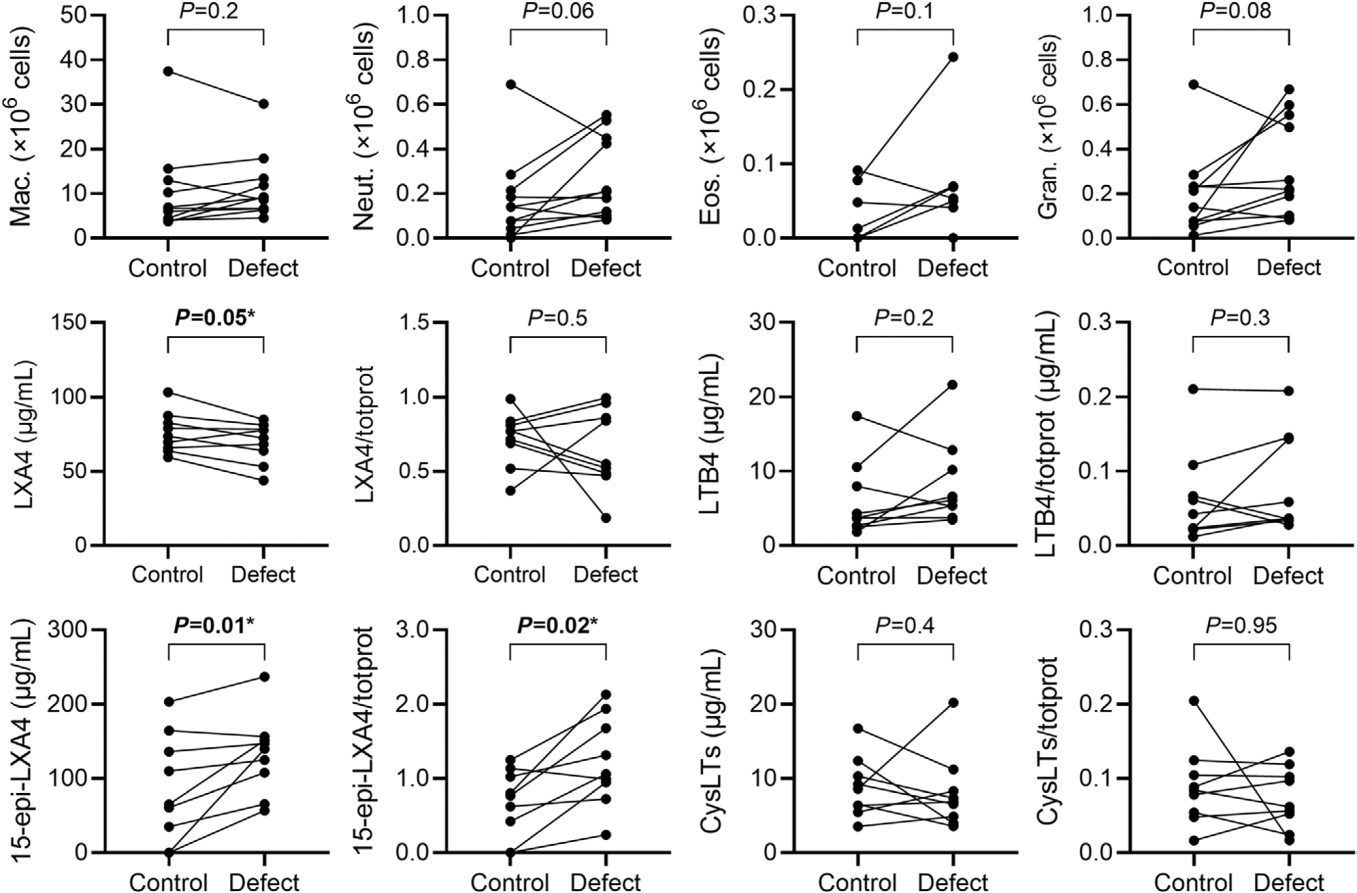
Bronchoalveolar Lavage (BAL) Cells and Lipid Proteins. Differential cell and lipid and protein levels from BAL samples in paired control versus defect sites. There were trends to increases in neutrophil (*p* = 0.06) and total granulocyte counts (*p* = 0.08) in regions of defect versus controls. Immunomodulatory lipid proteins, including LXA4 was reduced in defect versus control sites (*p* = 0.05), while both 15‐epi‐LXA4 (*p* = 0.01) and 15‐epi‐LXA4/totprot (*p* = 0.02) were significantly increased. 15‐epi, 15‐epimer; CysLTs, cysteinyl leukotrienes; Eos, eosinophils; Gran, total granulocytes; LTB4, leukotriene B4; LXA4, eicosanoid lipoxin A4; Mac, macrophages; Neut, neutrophils; totprot, normalized to total protein concentration.

Figure 4 shows differences in cell counts and oxylipin mediators from BAL fluid. Neutrophil (defect: 0.27 ± 0.18 × 10^6^ cells, control: 0.17 ± 0.19 × 10^6^ cells; *p* = 0.06) and total granulocyte counts (defect: 0.32 ± 0.22 × 10^6^ cells, control: 0.19 ± 0.19 × 10^6^ cells; *p* = 0.08) had a trend for a difference in defect as compared to control sites; eosinophil cell counts were not different between sites (*p* = 0.1). Figure 4 further shows that eicosanoid lipoxin A4 (LXA4) was significantly less (defect: 69 ± 14 μg/mL, control: 76 ± 14 μg/mL; *p* = 0.05) while both 15‐epimer (15‐epi) of LXA4 (defect: 141 ± 52 μg/mL, control: 111 ± 61 μg/mL; *p* = 0.04) and 15‐epi‐LXA4 normalized to the total protein concentration (defect: 1.4 ± 0.5, control: 0.9 ± 0.3; *p* = 0.02) were significantly greater in defect compared to control sites. No differences were observed for leukotriene B4 (chemotactic for neutrophils) or cysteinyl leukotrienes (pro‐inflammatory oxylipins made by eosinophils) nor were there any differences noted for several pro‐inflammatory cytokines in the BAL (inclusive of Eotaxin, IFNg, IL‐13, IL‐5, and MCP‐1; data not shown).

**FIGURE 5:**
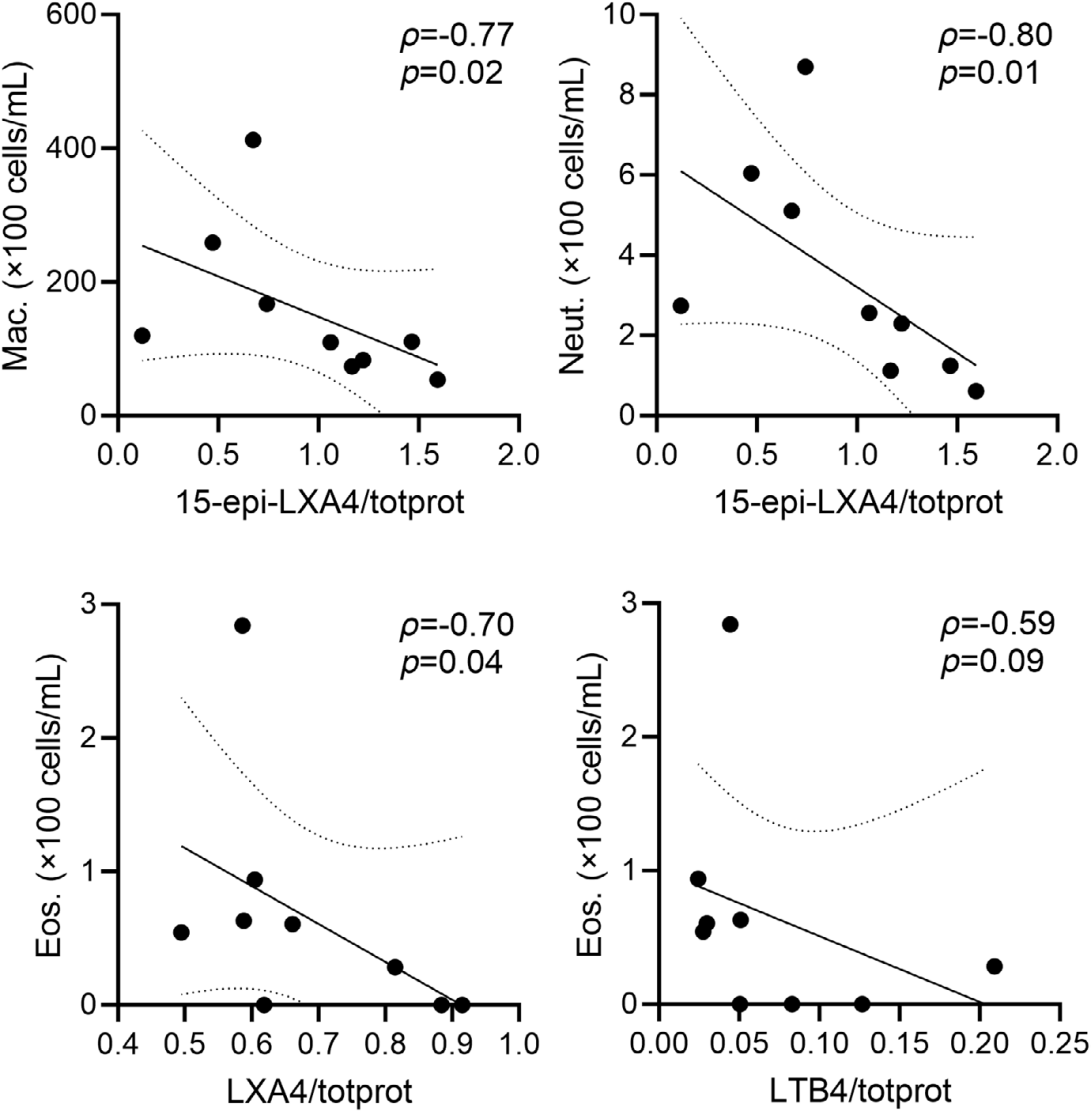
Relationships for average cell and lipid concentrations. Top left: BAL macrophage concentration inversely correlated with 15‐epi‐LXA4/totprot (*ρ* = −0.77, *p* = 0.03). Top right: Neutrophils inversely correlated with 15‐epi‐LXA4/totprot (*ρ* = −0.80, *p* = 0.01). Bottom left: Eosinophils inversely correlated with LXA4/totprot (*ρ* = −0.70, *p* = 0.04). Bottom right: There was a trend to an inverse correlation between eosinophils and LTB4/totprot (*ρ* = −0.59, *p* = 0.09). 15‐epi, 15‐epimer; Eos, eosinophils; Gran, total granulocytes; LTB4, leukotriene B4; LXA4, eicosanoid lipoxin A4; Mac, macrophages; Neut, neutrophils; totprot, normalized to total protein concentration.

Relationships between pro‐resolving oxylipins and granulocyte counts are presented in Figure 5. We observed inverse correlations between average BAL eosinophil concentration with normalized LXA4 (*ρ* = −0.70, *p* = 0.04), and between the average normalized 15‐epi‐LXA4 with neutrophils (*ρ* = −0.80, *p* = 0.01) and macrophages (*ρ* = −0.77, *p* = 0.02); average eosinophils and normalized leukotriene B4 trended towards an association (*ρ* = −0.59, *p* = 0.09).

### 3.2 Biopsy findings

**FIGURE 6:**
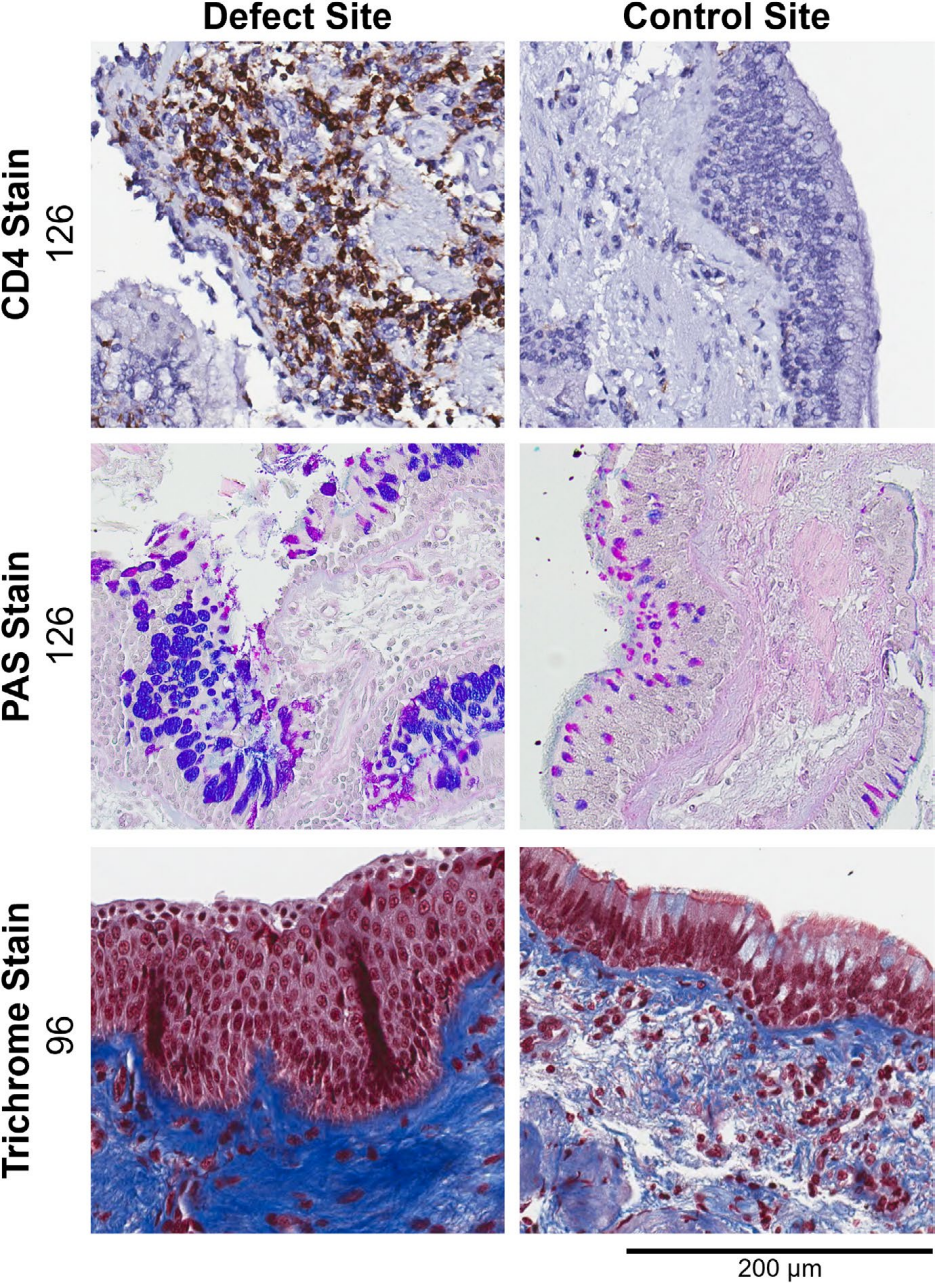
Paired Biopsy Samples from ventilation defect site (left) and control site (right) in the same participant. Top row: Samples from participant 126 (mild asthma) stained for CD4, which is preponderant in defect versus control samples. Middle row: Samples from participant 126 stained with Alcian Blue—Periodic Acid Schiff (PAS). Mucin appears pink and purple. Note increase in mucin and goblet cell hyperplasia in defect versus controls samples. Bottom row: Samples from participant 96 (no prior lung disease) stained with trichrome. Note stratified epithelium with squamous metaplasia and highlighting of collagen (blue), in defect versus control samples. Original magnification ×20 on all images.

Figure 6 shows CD4, Periodic Acid‐Schiff (PAS), and Masson's Trichrome stains of paired defect‐control biopsy samples in two participants. The CD4 stain shows a substantial increase in CD4 cells (stained brown) in defect versus control site. PAS shows increased mucin and goblet cell hyperplasia (blue and purple) in the defect site. Finally, Trichrome stain shows qualitative evidence of squamous metaplasia, loss of epithelial cilia, and collagen deposition (blue) characteristic of chronic airway injury and fibrosis. Basement membrane wall thickness was similar for tissue biopsies from defect versus control sites (6.97 ± 1.71 microns vs. 6.43 ± 2.54 microns, respectively; *p* = 0.9). Both participant 42 and 126 had evidence of elevated CD8, with participant 126 also demonstrating elevated CD4, CD10, and CD68, in defect as compared to control sites. There were no observable differences for participant 96 and 110 between defect and control sites for the cell marker stains evaluated.

## 4 DISCUSSION

We performed a novel image‐guided bronchoscopy approach using HP ^3^He MRI to sample sites of ventilation defect paired with well‐ventilated control sites within the lungs of the same patient in a pilot cohort of patients who had recently experienced a viral‐induced exacerbation. Prospectively identified areas of ventilation defects which are associated with airway inflammation and obstruction exhibited qualitative evidence of goblet cell hyperplasia and squamous metaplasia; trends suggesting greater numbers of neutrophils and granulocytes in defect regions; and significant differences in immune cell modifying proteins in defect regions compared to well‐ventilated control regions. In some instances, *p*‐values suggest mean differences that do not meet the traditional *p* < 0.05 threshold. These trends are maintained and reported to guide future work as our study is almost certainly underpowered due to the challenges of recruiting patients to imaging and bronchoscopy within a window that is shortly after viral‐induced asthma exacerbation. Future studies may benefit from further study of these trends.

Lipoxin protein levels including LXA4 and 15‐epi‐LXA4 were significantly different in defect versus control sites. Oxylipin mediators are derived from plasma cell membranes and contribute to the transition between pro‐inflammatory and pro‐resolution states (Tavares et al., 2023; Townsend et al., 2023). Lipoxins play an important role in the downregulation of inflammation, with 15‐epi‐LXA4 having been previously shown to facilitate the resolution of acute lung inflammation (Sekheri et al., 2020). In addition, the number of neutrophils and granulocytes trended towards an increase in defect regions. The presence of granulocytes in asthmatic airways is linked to increased disease severity and airway inflammation, and exacerbations (Fahy, 2009). Together, these findings are consistent with the immunomodulatory role of these proteins and strengthen earlier retrospective results linking ventilation defects with local inflammatory response (Fain et al., 2008).

In a subset of 4 participants, we conducted extensive analysis of a suite of HC and IHC markers of inflammation, injury, and cellular proliferation. In one such participant, PAS staining exhibited a visually striking increase in mucin and goblet cell hyperplasia in the defect region that included quantitative increases in CD4, CD8, CD10, and CD68 staining in the same biopsy sample. The presence of CD4 or CD8 cells in bronchial wall biopsies of asthma patients has been linked to a longitudinal loss of lung function measured via spirometry (den Otter et al., 2016). Another participant demonstrated an obviously stratified epithelial layer with squamous metaplasia and a marked increase in collagen in the tissue deep to the basement membrane in the tissue biopsy from the defect site, alterations which are characteristic of chronic airway injury and repair. Interestingly, this participant showed no observable differences in quantitative stains for cellular activity from the same site, which suggests that the observed pathophysiology in this participant may be the result of past chronic airway injury and inflammation.

The difference in inflammatory cell counts and mediators between defect and control sites as well as evidence of abnormal pathology within defect regions demonstrate the utility of functional image‐guided bronchoscopy, suggesting the potential for image‐guided treatment interventions. In fact, asthma patients treated using image‐guided bronchial thermoplasty using HP gas MRI (Hall et al., 2020; Svenningsen et al., 2021) have similar improvements in quality of life as compared to patients treated using standard bronchial thermoplasty protocols, but significantly reduced number of radiofrequency activations. Future clinical practice which combines the ability to identify and localize abnormally functioning airway segments and evaluate the underlying pathology to treat with phenotype‐specific regimens and image‐guided interventions could result in improved outcomes in asthma patients.

Important limitations to this work include the small sample size, especially in the *N* = 4 subset of participants in which HC/IHC staining was performed. Histology sections were further limited in sample size in this study given the airway epithelium integrity was not intact in most of the biopsy samples acquired. Moreover, such qualitative differences are also prone to sampling bias within a given tissue section. Although the generalizability of these findings is limited, there is still value in comparing paired defect and control sites for sections where control and defect epithelium are both intact as shown in Figure 6. More robust methods of biopsy tissue sampling and handling are currently being used for ongoing studies in severe asthma. In addition, HP ^3^He MRI was not acquired in three participants and thus, regions of CT air trapping were chosen as defect sites. Previous work, however, has demonstrated a spatial relationship between MRI ventilation defects and CT air trapping (Carey et al., 2023; Fain et al., 2008), hence, regions of air trapping identified using CT are likely a good estimate and surrogate for ^3^He MRI ventilation defect regions. Hyperpolarized MRI studies in the lungs have also largely replaced ^3^He gas with ^129^Xe gas, which has slightly different volumes of distribution that could have small but measurable effects on airway defect size (Stewart et al., 2018). Finally, as MRI and CT air trapping were acquired only following an exacerbation, it is difficult to discern whether the ventilation defects selected for image‐guided bronchoscopy in these patients were related to active airway inflammation in response to the viral‐induced exacerbation or whether they were the consequence of prior airway injury. We would also like to note that this study was performed before CT‐image guided bronchoscopy was implemented into routine clinical practice.

### 4.1 Conclusion

In this study, we have demonstrated the feasibility and utility of bronchoscopy guided by functional imaging. In 11 participants who had recently experienced a virus‐induced exacerbation, lung regions with abnormal airway function identified via HP gas MRI exhibited an increased presence of inflammatory cells and mediators as compared to well‐functioning lung regions. Results in this pilot population suggest that ventilation patterns observed via HP gas MRI provide a valuable tool for the identification and evaluation of localized airway phenomena associated with regional reductions in lung function. Advancing the understanding of airway activity specifically associated with localized regions of airway obstruction in future investigations may help refine the definition of asthma phenotypes and guide the development and evaluation of personalized, targeted therapies.

## AUTHOR CONTRIBUTIONS

DGM was responsible for developing the image guidance workflow, image processing, analysis, and interpretation and for preparing the first draft of the manuscript. KJC was responsible for data collection, image analysis and pathology interpretation. MJM was responsible for statistical analysis, interpretation of the results and writing and editing the manuscript. SK was responsible for data collection and biopsy sample analysis. SE was responsible for developing methodology and recommended cell‐marker stains for tissue pathology, and for processing and analysis of BAL lipid protein analysis. MWJ was responsible for developing methodology and recommended cell‐marker stains for tissue pathology, and interpretation of the results. MDE was responsible for statistical analysis and design. RLS was responsible for developing the study design and interpretation of the results. MS was responsible for interpretation of the results. NNJ was responsible for developing the study design, supervising the bronchoscopy protocol and interpretation of the results. LCD was responsible for developing the study design, performing bronchoscopy procedures and interpretation of the results. SBF was responsible for developing the study design, interpretation of the results, and direction of all aspects of the study, and writing and editing the manuscript. All authors had an opportunity to review and revise the manuscript and approved its final submitted version.

## FUNDING INFORMATION

This study was funded by National Heart, Lung, and Blood Institute (NHLBI) grant R01 HL080412 (Jarjour) with institutional resources funded by National Center for Advancing Translational Sciences (NCATS) grant 1 UL1RR025011. Secondary analyses were supported by R01 HL115118 (Denlinger) with effort contributed by The William W. and Judith H. Busse Professorship in Allergy and Asthma Research. SBF is supported by the National Institutes of Health (NIH) R01 HL126771, R01 HL146689, and R01 HL169765.

## CONFLICT OF INTEREST STATEMENT

DGM and SBF serve as scientific advisors for Polarean LLC. SBF also receives research funding from Polarean LLC, Siemens Healthineers INC, and GE Healthcare INC for development of pulmonary CT and MRI methods. MWJ has received research funding from Hoffmann‐La Roche (unrelated to this work). LCD reports consultation with GlaxoSmithKline, AstraZeneca, Sanofi, and OM Pharma during the course of this study. MJM, KJC, SK, SE, MDE, RLS, MS and NNJ have no disclosures.

## 5 ETHICS STATEMENT

This human study was approved by University of Wisconsin Health Sciences Institutional Review Board ‐ approval: (IRB H‐2005‐0070). All participants provided written informed consent to participate in this study.

## Data Availability

Data measures and analysis results can be made available by the corresponding author by request.

## References

[phy270119-bib-0001] Aysola, R. , de Lange, E. E. , Castro, M. , & Altes, T. A. (2010). Demonstration of the heterogeneous distribution of asthma in the lungs using CT and hyperpolarized helium‐3 MRI. Journal of Magnetic Resonance Imaging, 32, 1379–1387. 21105142 10.1002/jmri.22388

[phy270119-bib-0002] Bonnans, C. , Gras, D. , Chavis, C. , Mainprice, B. , Vachier, I. , Godard, P. , & Chanez, P. (2007). Synthesis and anti‐inflammatory effect of lipoxins in human airway epithelial cells. Biomedicine & Pharmacotherapy, 61, 261–267. 17418999 10.1016/j.biopha.2007.02.016

[phy270119-bib-0003] Carey, K. J. , Hotvedt, P. , Mummy, D. G. , Lee, K. E. , Denlinger, L. C. , Schiebler, M. L. , Sorkness, R. L. , Jarjour, N. N. , Hatt, C. R. , Galban, C. J. , & Fain, S. B. (2023). Comparison of hyperpolarized (3)He‐MRI, CT based parametric response mapping, and mucus scores in asthmatics. Frontiers in Physiology, 14, 1178339. 37593238 10.3389/fphys.2023.1178339 PMC10431597

[phy270119-bib-0004] Crooks, S. W. , & Stockley, R. A. (1998). Leukotriene B4. The International Journal of Biochemistry & Cell Biology, 30, 173–178. 9608670 10.1016/s1357-2725(97)00123-4

[phy270119-bib-0005] den Otter, I. , Willems, L. N. , van Schadewijk, A. , van Wijngaarden, S. , Janssen, K. , de Jeu, R. C. , Sont, J. K. , Sterk, P. J. , & Hiemstra, P. S. (2016). Lung function decline in asthma patients with elevated bronchial CD8, CD4 and CD3 cells. The European Respiratory Journal, 48, 393–402. 27230446 10.1183/13993003.01525-2015

[phy270119-bib-0006] Denlinger, L. C. , Sorkness, R. L. , Lee, W. M. , Evans, M. D. , Wolff, M. J. , Mathur, S. K. , Crisafi, G. M. , Gaworski, K. L. , Pappas, T. E. , Vrtis, R. F. , Kelly, E. A. , Gern, J. E. , & Jarjour, N. N. (2011). Lower airway rhinovirus burden and the seasonal risk of asthma exacerbation. American Journal of Respiratory and Critical Care Medicine, 184, 1007–1014. 21816938 10.1164/rccm.201103-0585OC PMC3208645

[phy270119-bib-0007] Eddy, R. L. , Svenningsen, S. , Licskai, C. , McCormack, D. G. , & Parraga, G. (2019). Hyperpolarized helium 3 MRI in mild‐to‐moderate asthma: Prediction of postbronchodilator reversibility. Radiology, 293, 212–220. 31385758 10.1148/radiol.2019190420

[phy270119-bib-0008] Fahy, J. V. (2009). Eosinophilic and neutrophilic inflammation in asthma: Insights from clinical studies. Proceedings of the American Thoracic Society, 6, 256–259. 19387026 10.1513/pats.200808-087RM

[phy270119-bib-0009] Fain, S. B. , Gonzalez‐Fernandez, G. , Peterson, E. T. , Evans, M. D. , Sorkness, R. L. , Jarjour, N. N. , Busse, W. W. , & Kuhlman, J. E. (2008). Evaluation of structure‐function relationships in asthma using multidetector CT and hyperpolarized He‐3 MRI. Academic Radiology, 15, 753–762. 18486011 10.1016/j.acra.2007.10.019 PMC2744977

[phy270119-bib-0010] Godard, P. , Chaintreuil, J. , Damon, M. , Coupe, M. , Flandre, O. , Crastes de Paulet, A. , & Michel, F. B. (1982). Functional assessment of alveolar macrophages: Comparison of cells from asthmatics and normal subjects. The Journal of Allergy and Clinical Immunology, 70, 88–93. 7096825 10.1016/0091-6749(82)90234-2

[phy270119-bib-0011] Hall, C. S. , Quirk, J. D. , Goss, C. W. , Lew, D. , Kozlowski, J. , Thomen, R. P. , Woods, J. C. , Tustison, N. J. , Mugler, J. P., 3rd , Gallagher, L. , Koch, T. , Schechtman, K. B. , Ruset, I. C. , Hersman, F. W. , & Castro, M. (2020). Single‐session bronchial Thermoplasty guided by (129)Xe magnetic resonance imaging. A Pilot Randomized Controlled Clinical Trial. American Journal of Respiratory and Critical Care Medicine, 202, 524–534. 32510976 10.1164/rccm.201905-1021OC PMC7427382

[phy270119-bib-0012] Kauczor, H. U. , Ebert, M. , Kreitner, K. F. , Nilgens, H. , Surkau, R. , Heil, W. , Hofmann, D. , Otten, E. W. , & Thelen, M. (1997). Imaging of the lungs using 3He MRI: Preliminary clinical experience in 18 patients with and without lung disease. Journal of Magnetic Resonance Imaging, 7, 538–543. 9170039 10.1002/jmri.1880070314

[phy270119-bib-0013] Kelly, E. A. , Esnault, S. , Liu, L. Y. , Evans, M. D. , Johansson, M. W. , Mathur, S. , Mosher, D. F. , Denlinger, L. C. , & Jarjour, N. N. (2017). Mepolizumab attenuates airway eosinophil numbers, but not their functional phenotype, in asthma. American Journal of Respiratory and Critical Care Medicine, 196, 1385–1395. 28862877 10.1164/rccm.201611-2234OC PMC5736971

[phy270119-bib-0014] Lozewicz, S. , Wells, C. , Gomez, E. , Ferguson, H. , Richman, P. , Devalia, J. , & Davies, R. J. (1990). Morphological integrity of the bronchial epithelium in mild asthma. Thorax, 45, 12–15. 2321171 10.1136/thx.45.1.12 PMC475632

[phy270119-bib-0015] Mathew, L. , Evans, A. , Ouriadov, A. , Etemad‐Rezai, R. , Fogel, R. , Santyr, G. , McCormack, D. G. , & Parraga, G. (2008). Hyperpolarized 3He magnetic resonance imaging of chronic obstructive pulmonary disease: Reproducibility at 3.0 tesla. Academic Radiology, 15, 1298–1311. 18790402 10.1016/j.acra.2008.04.019

[phy270119-bib-0016] Mets, O. M. , van Hulst, R. A. , Jacobs, C. , van Ginneken, B. , & de Jong, P. A. (2012). Normal range of emphysema and air trapping on CT in young men. AJR. American Journal of Roentgenology, 199, 336–340. 22826394 10.2214/AJR.11.7808

[phy270119-bib-0017] Moller, H. E. , Chen, X. J. , Saam, B. , Hagspiel, K. D. , Johnson, G. A. , Altes, T. A. , de Lange, E. E. , & Kauczor, H. U. (2002). MRI of the lungs using hyperpolarized noble gases. Magnetic Resonance in Medicine, 47, 1029–1051. 12111949 10.1002/mrm.10173

[phy270119-bib-0018] Moore, W. C. , Evans, M. D. , Bleecker, E. R. , Busse, W. W. , Calhoun, W. J. , Castro, M. , Chung, K. F. , Erzurum, S. C. , Curran‐Everett, D. , Dweik, R. A. , Gaston, B. , Hew, M. , Israel, E. , Mayse, M. L. , Pascual, R. M. , Peters, S. P. , Silveira, L. , Wenzel, S. E. , Jarjour, N. N. , & National Heart L, and Blood Institute's Severe Asthma Research G . (2011). Safety of investigative bronchoscopy in the severe asthma research program. The Journal of Allergy and Clinical Immunology, 128, 328–336. e323. 21496892 10.1016/j.jaci.2011.02.042 PMC3149754

[phy270119-bib-0019] Mummy, D. , Kehoe, S. , Aesif, S. , Sorkness, R. , Jarjour, N. , Schiebler, M. , Evans, M. , Denlinger, L. , & Fain, S. (2018). Image‐guided bronchoscopy of regional ventilation heterogeneity in asthma as a means of assessing localized inflammatory response: Preliminary results. American Journal of Respiratory and Critical Care Medicine, 197, A2745.

[phy270119-bib-0020] Mummy, D. , Sorkness, R. , Denlinger, L. , Jarjour, N. , & Fain, S. (2015). Magnetic resonance image‐guided Bronchoscopic sampling in asthma indicates increased levels of granulocytes in areas of hyperpolarized 3he ventilation defect compared with control sites. American Journal of Respiratory and Critical Care Medicine, 191, A6143.

[phy270119-bib-0021] Mummy, D. G. , Carey, K. J. , Evans, M. D. , Denlinger, L. C. , Schiebler, M. L. , Sorkness, R. L. , Jarjour, N. N. , & Fain, S. B. (2020). Ventilation defects on hyperpolarized helium‐3 MRI in asthma are predictive of 2‐year exacerbation frequency. The Journal of Allergy and Clinical Immunology, 146, 831–839. e836. 32173351 10.1016/j.jaci.2020.02.029 PMC7487001

[phy270119-bib-0022] Mummy, D. G. , Kruger, S. J. , Zha, W. , Sorkness, R. L. , Jarjour, N. N. , Schiebler, M. L. , Denlinger, L. C. , Evans, M. D. , & Fain, S. B. (2018). Ventilation defect percent in helium‐3 magnetic resonance imaging as a biomarker of severe outcomes in asthma. The Journal of Allergy and Clinical Immunology, 141, 1140–1141. e1144. 29129582 10.1016/j.jaci.2017.10.016 PMC5844809

[phy270119-bib-0023] Niles, D. J. , Kruger, S. J. , Dardzinski, B. J. , Harman, A. , Jarjour, N. N. , Ruddy, M. , Nagle, S. K. , Francois, C. J. , & Fain, S. B. (2013). Exercise‐induced bronchoconstriction: Reproducibility of hyperpolarized 3He MR imaging. Radiology, 266, 618–625. 23169798 10.1148/radiol.12111973 PMC5411018

[phy270119-bib-0024] Pike, D. , Kirby, M. , Guo, F. , McCormack, D. G. , & Parraga, G. (2015). Ventilation heterogeneity in ex‐smokers without airflow limitation. Academic Radiology, 22, 1068–1078. 26008133 10.1016/j.acra.2015.04.006

[phy270119-bib-0025] Sekheri, M. , El Kebir, D. , Edner, N. , & Filep, J. G. (2020). 15‐epi‐LXA(4) and 17‐epi‐RvD1 restore TLR9‐mediated impaired neutrophil phagocytosis and accelerate resolution of lung inflammation. Proceedings of the National Academy of Sciences of the United States of America, 117, 7971–7980. 32205444 10.1073/pnas.1920193117 PMC7149425

[phy270119-bib-0026] Sieren, J. P. , Newell, J. D., Jr. , Barr, R. G. , Bleecker, E. R. , Burnette, N. , Carretta, E. E. , Couper, D. , Goldin, J. , Guo, J. , Han, M. K. , Hansel, N. N. , Kanner, R. E. , Kazerooni, E. A. , Martinez, F. J. , Rennard, S. , Woodruff, P. G. , Hoffman, E. A. , & Group SR . (2016). SPIROMICS protocol for multicenter quantitative computed tomography to phenotype the lungs. American Journal of Respiratory and Critical Care Medicine, 194, 794–806. 27482984 10.1164/rccm.201506-1208PP PMC5074650

[phy270119-bib-0027] Stewart, N. J. , Chan, H. F. , Hughes, P. J. C. , Horn, F. C. , Norquay, G. , Rao, M. , Yates, D. P. , Ireland, R. H. , Hatton, M. Q. , Tahir, B. A. , Ford, P. , Swift, A. J. , Lawson, R. , Marshall, H. , Collier, G. J. , & Wild, J. M. (2018). Comparison of (3) He and (129) Xe MRI for evaluation of lung microstructure and ventilation at 1.5T. Journal of Magnetic Resonance Imaging, 48, 632–642. 29504181 10.1002/jmri.25992 PMC6175321

[phy270119-bib-0028] Svenningsen, S. , Eddy, R. L. , Lim, H. F. , Cox, P. G. , Nair, P. , & Parraga, G. (2018). Sputum eosinophilia and magnetic resonance imaging ventilation heterogeneity in severe asthma. American Journal of Respiratory and Critical Care Medicine, 197, 876–884. 29313707 10.1164/rccm.201709-1948OC

[phy270119-bib-0029] Svenningsen, S. , Nair, P. , Eddy, R. L. , McIntosh, M. J. , Kjarsgaard, M. , Lim, H. F. , McCormack, D. G. , Cox, G. , & Parraga, G. (2021). Bronchial thermoplasty guided by hyperpolarised gas magnetic resonance imaging in adults with severe asthma: A 1‐year pilot randomised trial. ERJ Open Res, 7. 10.1183/23120541.00268-2021 PMC8473812 34589541

[phy270119-bib-0030] Takano, T. , Fiore, S. , Maddox, J. F. , Brady, H. R. , Petasis, N. A. , & Serhan, C. N. (1997). Aspirin‐triggered 15‐epi‐lipoxin A4 (LXA4) and LXA4 stable analogues are potent inhibitors of acute inflammation: Evidence for anti‐inflammatory receptors. The Journal of Experimental Medicine, 185, 1693–1704. 9151906 10.1084/jem.185.9.1693 PMC2196289

[phy270119-bib-0031] Tavares, L. P. , Nijmeh, J. , & Levy, B. D. (2023). Respiratory viral infection and resolution of inflammation: Roles for specialized pro‐resolving mediators. Experimental Biology and Medicine (Maywood, N.J.), 248, 1635–1644. 10.1177/15353702231199082 PMC10723024 37837390

[phy270119-bib-0032] Townsend, E. A. , Guadarrama, A. , Shi, L. , Roti Roti, E. , & Denlinger, L. C. (2023). P2X(7) signaling influences the production of pro‐resolving and pro‐inflammatory lipid mediators in alveolar macrophages derived from individuals with asthma. American Journal of Physiology. Lung Cellular and Molecular Physiology, 325, L399–L410. 37581221 10.1152/ajplung.00070.2023 PMC10639011

[phy270119-bib-0033] Zha, W. , Kruger, S. J. , Cadman, R. V. , Mummy, D. G. , Evans, M. D. , Nagle, S. K. , Denlinger, L. C. , Jarjour, N. N. , Sorkness, R. L. , & Fain, S. B. (2018). Regional heterogeneity of lobar ventilation in asthma using hyperpolarized Helium‐3 MRI. Academic Radiology, 25, 169–178. 29174189 10.1016/j.acra.2017.09.014 PMC6554715

